# Differences in visual fixation duration according to the position of graphic health warning labels: An eye-tracking approach

**DOI:** 10.18332/tid/94327

**Published:** 2018-09-04

**Authors:** Ji-eun Hwang, Yu-seon Yang, Yu-mi Oh, Seon-young Lee, Joung-eun Lee, Sung-il Cho

**Affiliations:** 1 Department of Public Health Science, Graduate School of Public Health, Seoul National University, Seoul, Republic of Korea; 2 Korea Health Promotion Institute, Seoul, Republic of Korea; 3 Gyeonggi Infectious Disease Control Center, Seongnam, Republic of Korea; 4 Institute of Health and Environment, Seoul National University, Seoul, Republic of Korea

**Keywords:** tobacco control, graphic health warning label, FCTC, eye tracking

## Abstract

**INTRODUCTION:**

The Framework Convention on Tobacco Control (FCTC) recommends that graphic health warning labels (GHWLs) be positioned at the top of the principal area of cigarette packs, rather than at the bottom, to increase visibility. However, during the legislative process of introducing GHWLs in South Korea, the position of GHWLs has become a contested issue. The pro-tobacco industry group argued that the warnings should be placed at the bottom of cigarette packs because evidence for the effectiveness of the upper position was insufficient. Therefore, this study investigated whether the position of the GHWL affects eye movement.

**METHODS:**

Participants (30 daily smokers and 24 non-smokers) were shown six cigarette packs in random order with different position combinations (top, middle, bottom) and image concepts (skin aging, toxic constituents). Participants’ eye movements were recorded using eye-tracking equipment to measure visual fixation duration in milliseconds (ms)

**RESULTS:**

Participants visually fixated longer on the health warning area than on the tobacco branding area (p<0.05). Mean fixation duration on the health warning area was significantly longer at the top or middle positions compared to the bottom, by 28% (mean difference=340 ms, p=0.006) and by 30% (mean difference=368 ms, p=0.002), respectively. By contrast, mean fixation duration on the branding area was longer with the warning at the bottom compared to top or middle positions by 25% and 33%, with mean differences of 157 ms (p=0.100) and 212 ms (p=0.026), respectively. No significant difference in fixation time was observed between the top and middle positions (p>0.05)

**CONCLUSIONS:**

The duration of visual fixation on GHWLs was longer when they were displayed at the top and middle, rather than at the bottom. Therefore, GHWLs should be positioned from the top to the middle of the tobacco package.

## INTRODUCTION

The use of graphic health warning labels (GHWLs) is an effective policy for informing and persuading smokers and non-smokers about the negative consequences associated with tobacco products and smoking^1,2^. GHWLs not only promote smoking cessation among smokers, but also are effective in preventing smoking initiation among non-smokers, especially young people^3,4^.

For this reason, the World Health Organization Framework Convention on Tobacco Control (FCTC) recommends that each Party introduces a warning, preferably with the use of pictures, within 3 years after entry into force of the Convention for that Party (Article 11)^5,6^. The guidelines recommend the use of certain design elements, including warnings on the front and back, coverage of ≥ 50% of the principal package area, and periodic revision of the health warnings to enhance their effectiveness^6^. To increase the visibility, the guidelines also require that health warnings be positioned at the top of the principal area rather than at the bottom^6^. However, some countries continue to place warning signs at the bottom of the packaging^7^.

In South Korea, GHWLs must be placed at the top of both the front and back of tobacco packaging, as of 23 December 2016^8^. However, the position of GHWLs became a major issue during the legislative process related to the introduction of GHWLs in South Korea^9,10^. Pro-tobacco industry groups argued that the warnings should be placed at the bottom of cigarette packs because the effectiveness of the upper position was not supported by sufficient evidence^9,10^.

The placement of a warning label influences the likelihood that it is noticed and understood^11^. Therefore, effective warning positioning may draw the consumers’ attention to relevant information for more healthy choices^11^. In general, when viewing an object, eye-movement is controlled both by top-to-bottom and bottom-to-top processes affecting attention^12^. Specifically, according to the Gutenberg diagram, the viewer is likely to pay more attention to the elements at the top-left than at the bottomright^13^. This top-to-bottom layout is related to the process of visual recognition based on the task or intention^14^; it follows that this layout could influence decision making^12^. Thus, it is recommended that various warning labels be printed at the top of the principal areas (e.g. nutrition labels, warnings for alcohol, other products)^15-17^.

Although a variety of studies have been conducted examining various aspects of warning design and how they affect attitude, knowledge and behavior, to date few studies have demonstrated the effects of the positioning of GHWLs at the top, middle, or bottom of packaging. As previous studies measured subjective attitudes and did not include experimental manipulations related to the position of the warning^18,19^, our ability to directly compare the effects of differently positioned GHWLs is limited. Additionally, the majority of existing experimental studies have compared responses to text about and pictures of tobacco products^20^.

Experimental studies, such as those involving eye tracking, overcome the unreliability of self-reports of particular attitudes and behaviors of interest^21^. Measurements of visual fixation duration can be interpreted in the context of information processing, recall, and attention, making the results of eye-tracking studies particularly relevant to the strengthening of tobacco control policies^20^.

Therefore, this study used the eye-tracking method to investigate whether the position of GHWLs affects eye movements. Based on previous literature^12,15,16^, we hypothesized that fixation time would differ according to the position of the health warning. Additionally, we expected these differences to depend on the concept of the warning image and smoking status. Previous experimental studies using eye tracking to measure response to GHWL obtained different results, depending on respondents’ characteristics and warning image features^20^.

## METHODS

### Design

This study used a cross-over design with health warning position (top, middle, bottom) and warning image concept (skin aging, toxic constituents) as within-subject factors, and smoking status (smoker, non-smoker) as between-subject factors. In cross-over trials design each subject receives a sequence of experimental treatments^22^. In our study, each participant viewed a total of six cigarette packs (three positions × two image concepts), with the order of presentation randomized for each participant to mitigate order effects. Eye-tracking equipment was used to measure the fixation duration of eye movements towards both graphic health warnings and branding on cigarette packs.

### Participants

Participants were recruited for this study by a professional research institution (Brain & Research Inc., Seoul, South Korea). The institution randomly sent a recruitment e-mail to a panel of previously identified subjects. Candidates who responded to the e-mail were called in to screen for eligibility.

Employees of the cigarette manufacturing and production industry were excluded from this study. In addition, people with corrected vision under 0.5, severe astigmatism and strabismus, eyes too small to wear lenses, ophthalmic diseases, psychiatric disorders, and neurologic diseases were excluded. Non-smokers were defined as individuals who had not smoked more than 100 cigarettes in their lifetime and did not currently smoke; smokers were defined as individuals who had smoked at least 100 cigarettes in their lifetime and who currently smoked cigarettes. Smokers with a total smoking period of less than one year and non-smokers who intended to start smoking within the next year were excluded.

Considering gender and smoking status, we finally confirmed 54 participants for this study. Prior to the eye-tracking survey, self-reported questionnaires were used to measure age, gender, occupation, current disease status and current smoking status.

### Materials

Visual images were designed specifically for this study. Six photographs of cigarette packs were prepared with different combinations of three positions (top, middle, bottom) and two warning image concepts (skin aging, toxic constituents). The health warning images used for this study were part of 10 images developed by Sogang University (Seoul, South Korea) and the Korea Health Promotion Institute (Seoul, South Korea) in 2014 prior to GHWL implementation^23^. The photograph was presented at twice life size (110×174 versus 55×87 mm). The images displayed a health warning, which covered 50% of the display area, and branding, which covered 50% of the display area. For data analysis, areas of interest (AOIs), which were identified *a priori* for each image, consisted of the health warning (health warning AOI) and the branding (branding AOI). Thus, three different packaging layouts were created. In the first layout, the health warning was placed in the upper half of the packaging, with the branding on the bottom half. In the second layout, the health warning was placed in the middle, and the branding was divided between the top 25% and the bottom 25%. In the third layout, the branding occupied the top half of the packaging, and the warning was on the bottom half.

### Measures

Fixation duration measures the time that a participant’s gaze stays within a relatively specific area^24^, so this study measured fixation duration to analyze the effects of the specific position of health warnings placed on cigarette packages. Each warning image was presented automatically on the screen to participants for 3000 ms, and fixation duration on both the health and branding AOIs during this time was measured with minimum threshold of 100 ms.

In this study, fixation duration on the health warning AOI was defined as the total number of seconds the participant looked at the health warning AOI during a total of 3000 ms, and fixation duration on the branding AOI was defined as the total number of seconds the participant looked at the branding AOI during 3000 ms.

### Eye-tracking procedure

This study proceeded as follows: after an introduction to the experiment was provided, a preliminary survey to gather data on personal characteristics was administered; this was followed by an eye-tracking experiment to measure participants’ eye movements while they were viewing images of cigarette packages. Testing took place individually in Seoul, South Korea, between 22 November 2015 and 1 December 2015.

Eye tracking was conducted in an independent laboratory. The subjects rested their chin and forehead on the chin rest of the eye tracker located 50 cm away from the 27-inch color monitor with the level of resolution set at 1280×720 pixels while comfortably sitting on a chair in front of the desk where the monitor and eye tracker were placed. While viewing each image, which appeared to be randomly selected, the eye movements of the participants were recorded with eye-tracking equipment for 3000 ms whenever they focused on the AOI. A gray masking screen was inserted for 5000 ms between warnings to control for the carryover and order effects that can occur when a number of warnings are seen repeatedly^25,26^. Eye fixation duration was measured by EyeTribe tracker (The Eye Tribe, 2014). In this study, the accuracy required ranged from 0.5° to 1.0°, and the sampling rate was 30 Hz for 3000 ms.

### Data analysis

We analyzed a cross-over design with health warning position (top, middle, bottom) and image concept (skin aging, toxic constituents) as within-subject factors, and smoking status (smoker, non-smoker) as between-subject factors. Because of gender differences in the response to smoking-related cues, gender was included as a covariate^27,28^. The ANOVA tested the main effects of health warning position, warning image concept, smoking status, and their interactions. All the statistical analysis was performed using SPSS 24, and p-values <0.05 were considered significant.

## RESULTS

In total, 54 participants (30 males, 24 females) with a mean age of 32.31 years (standard deviation, SD=6.43) were recruited for this study. There were 15 smokers and 9 non-smokers in the female group, and 15 smokers and 15 non-smokers in the male group. The 30 smokers had low average nicotine dependency (2.33 points)^29^. Also, approximately half (n=30) of the participants were unemployed, 38.9% (n=21) were white collar workers and 5.6% (n=3) were blue collar. In addition, they had no ophthalmic diseases or neurologic diseases.

All participants viewed each of the six cigarette pack images for an average of 1851 ms (SD=689 ms), and participants fixated longer on the warning (M=1451 ms, SD=766 ms) than on the branding (M=689 ms, SD=464, p<0.001) when both were located in the AOI.

Table 1 shows the mean fixation duration of each AOI by health warning position. For all three health warning positions, participants spent a longer time looking at the warning AOI (top: M=1547 ms, SD=732; middle: M=1574 ms, SD=776; bottom: M=1207 ms, SD=743) than the branding AOI (top: M=639 ms, SD=381; middle: M=584 ms, SD=384; bottom: M=796 ms, SD=547), and the difference was statistically significant for all three health warning positions (top: t(68)=6.14, p<0.001; middle: t(51)=6.29, p<0.001; bottom: t(74)=3.22, p=0.002).

**Table 1:** Mean fixation duration on the health warning and branding areas of cigarette packages according to health warning position (n = 54)

	*Health warning position*			
	*Top*	*Middle*	*Bottom*	*p†*
Fixation duration (ms), mean (SD)				
Health warning AOI	1547 (732)	1574 (776)	1207 (743)	0.001**
Branding AOI	639 (381)	584 (384)	796 (547)	0.017*

ms: millisecond, SD: standard deviation, AOI: area of interest. †One-way ANOVA was applied to compare the mean fixation duration among the three positions (top, middle, bottom) by each AOI.

** p<0.01

* p<0.05

One-way ANOVA showed a significant difference in the mean fixation duration among the three positions for both the warning AOI (*F* [2, 291]=7.01, p=0.001, η^2^=0.046) and branding AOI (*F*[2, 208]=4.17, p=0.017, η^2^=0.039). A *post hoc* test using the Bonferroni method indicated that the mean fixation duration on the health warning AOI was significantly longer at the top or middle positions compared to the bottom, by 28% (mean difference=340 ms, p=0.006) and by 30% (mean difference=368 ms, p=0.002), respectively. By contrast, mean fixation duration on the branding AOI was longer with the warning at the bottom compared to top or middle positions by 25% and 33%, with mean differences of 157 ms (p=0.100) and 212 ms (p=0.026), respectively.

For the health warning, females showed a longer fixation duration than males (females: M=1577 ms, SD=723; males: M=1355 ms, SD=793; t(292)=2.27, p=0.024). Although not significant (p>0.05), smokers tended to have longer fixation durations on the health warning than non-smokers (smokers: M=1518 ms, SD=783; non-smokers: M=1359 ms, SD=735; t(292)=1.76, p=0.08).

For descriptive purposes, ANOVA was used to test the main effects of the within-subject factors health warning position (top, middle, bottom) and warning image concept (skin aging, toxic constituents) and the between-subject factor smoking status (smokers, non-smokers) on the fixation duration of the health warning. No significant three-way interactions were detected among the health warning position, warning image concept, and smoking status (*F*[2, 281]=1.82, p=0.164, η^2^=0.013). Additionally, there was no two-way interaction between the health warning position and smoking status (*F*[2, 281]=2.84, p=0.060, η^2^=0.020), between health warning position and warning image concept (*F*[2, 281]=0.68, p=0.505, η^2^=0.005), or between warning image concept and smoking status (*F*[1, 281]=2.70, p=0.102, η^2^=0.010).

**Figure 1:**
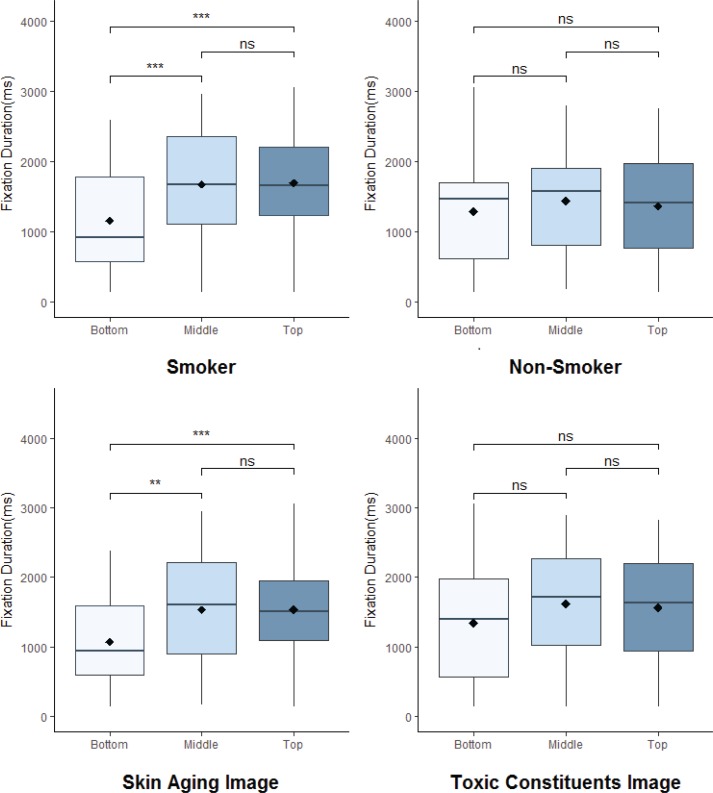
Fixation duration (in milliseconds) on the health warning according to position (top, middle, bottom) by smoking status and warning image type. Each box plot indicates the median (middle box line), the lower and upper quartiles (box ranges), and the mean (black diamond). **p<0.01, ***p<0.001, ns: non-significant.

However, as shown in Figure 1, the fixation duration was significantly longer when the health warning was placed at the top or the middle than at the bottom among smokers. The differences between the top and the bottom (t[108)]=3.89, p<0.001) and between the middle and the bottom were significant (t[110)]=3.58, p<0.001), but that between the top and the middle was not (t[116]=0.116, p=0.908). However, the fixation durations did not differ according to the position of the health warning among non-smokers (p>0.05).

The warning image concept affected fixation duration. For the skin aging image, the differences between the top and bottom positions (t[94]=3.43, p<0.001) and between the middle and bottom positions were significant (t[94]=3.24, p=0.002). However, for the toxic constituents’ image, the fixation duration did not differ according to the position of the health warning (p>0.05).

## DISCUSSION

The purpose of the present study was to investigate eye movements in response to GHWLs located at three different positions (top, middle, bottom) on cigarette packaging. As expected, fixation time depended on the position of the health warning. The difference in fixation duration on the health warning was significant between the top and bottom, middle and bottom, but not top and middle. Importantly, positioning at the top and middle increased by about 30% the duration of visual fixation on the GHWL. In addition, the mean fixation duration of the branding also differed according to the positioning. These indicate that the pro-tobacco industry’s claim that the position of warnings is irrelevant is incorrect.

According to Yarbus^30^, human eye movements respond to task context and stimulus content^30^. Repetitive fixation is the state in which the brain transmits a signal to fix the eyes, rather than the state in which the eye pupils stop and motion ceases^31^. Indeed, the pupils move continuously even when the gaze is fixed, and they cannot be fixed without brain function^31^. Therefore, the amount of information that can be cognitively processed increases with fixation duration^32^. In particular, viewing a product for a long period allows unconsciously perceived information to be processed and to affect the recall of the product^32^.

Placing a warning at the top or middle of packaging would capture the user’s attention for significantly longer time than warnings located at the bottom^15^. Because most consumers read by starting at the top, and they tend to prioritize the top and left-hand side of packaging, warnings placed in the upper portion were seen earlier than those placed at the bottom^15^. This top-to-bottom pattern is related to the process of visual recognition based on the task or intention^14^, furthermore, it influences decision making^12^. When visual attention is focused on a particular location, processing of that information is enhanced, and this contributes to memory and choice^33^.

In general, GHWLs capture and hold attention better than text warnings, and warning images and their messages could be recalled by smokers and non-smokers both short- and long-term^34-36^. We suggest that the increase in the fixation time on the upper or middle position of the health warning, compared to the bottom, demonstrated in this study that it could have a positive effect on the amount of information perceived and recall of the GHWL. Previous studies have shown such effects. In Canada, recall of one warning increased from 20% to 95% after the warning was moved from the bottom of the package to the top^18^. In Thailand, smokers’ awareness of health risks and their willingness to quit improved following the introduction of new warning labels that included graphic images placed in the top 50% of the package^19^. Therefore, it is desirable to arrange the GHWL at the top or middle instead of the bottom, and the GHWL should be enlarged to occupy most of the display area, so that distinguishing between the top and bottom becomes irrelevant.

Our results indicate that although positioning a health warning at the top or bottom of packaging affects visual attention, this effect differs according to smoking status and warning image concept. For smokers, visual attention response was relatively quick and sensitive to the warning position, but this was not true for non-smokers. Previous research has also shown that smokers pay more attention to graphic warnings than do non-smokers when viewing tobacco packaging^29,37,38^. In contrast to the warning text, the appeal of the warning picture, which was emphasized, was based on the loss frame^39^. Such loss-frame warnings elicit particularly negative emotional responses and are generally more effective among smokers than non-smokers^39,40^. This is because negative and loss-related messages are more influential than positive and gain-related messages when the contents of the message are strongly related to oneself^39,40^. Indeed, smokers likely fixed their visual attention on health warnings more than non-smokers because they thought the warning image on the cigarette package was related to them. However, in some studies^41,42^, smokers reacted defensively to high-risk images. Therefore, the effect of smoking status should continue to be considered depending on the risk level of warning images.

We found that design features of the warning affected responses. The skin aging image is likely easier to understand than the toxic constituents’ image, so participants seem to have focused more on the toxic constituents’ image. Because of the time to process information^32^, the toxic constituents’ image showed relatively minor differences depending on warning position. Therefore, future research should investigate whether warning images affect both smokers and non-smokers.

The top and middle positions of the GHWL were not only associated with increased visual attention to the warning but also enhanced exposure of the warning. Because GHWLs undermine the effect of a brand’s appeal and affect package displays at retail outlets^43,44^, the probability that a GHWL will be visible in stores increases when the GHWL is placed at the top. According to a survey of tobacco retail displays in Seoul, South Korea, before implementation of GHWLs^45^, the health warning texts placed at the bottom were hidden by the price tag and the product name of the cigarette. If GHWLs were placed at the bottom they would be covered by these attachments and would not be highly visible. Therefore, it is desirable to arrange the GHWLs at the middle and top, not at the bottom, to increase their visibility.

The current global trend is plain packaging, which standardizes all elements of tobacco product packaging^46^. Plain packaging, which began in 2012 in Australia, has now been introduced in the UK, France and Norway^47^. However, some countries have not introduced GHWLs yet, and there are only one or two warning images, or the warning area is <30%^7^. It is best to start with plain packaging, but if not, it is desirable to maximize the effect of the GHWL by the use of a larger area, borders, complementary colors, and rotation before introducing plain packaging^6^. In addition, the GHWL should be placed at the top or the middle of packaging to increase visual attention on the image. In countries where the image position remains at the bottom, it is recommended that the warnings be moved to the top or middle. In countries where GHWLs will be introduced in the future, efforts should be made to place warnings at the top or middle.

### Limitations

The present study has some limitations. First, only adults were surveyed. The GHWLs were created to prevent new smokers, such as youth and young adults from initiating smoking, so it will be necessary to conduct experiments on these cohorts as well. Second, it is difficult to objectively evaluate changes in behavior and perception according to the position of the GHWL by measuring fixation duration. If the interpretation of changes in cognition due to short- and long-term exposure is interpreted in conjunction with physiological results, the effect of position will be more objectively explained. Third, it is possible to arrange the images in the center of the screen in the same way during experimental research, as movement of the line of sight proceeds from the center. Fourth, because this study was conducted prior to the introduction of GHWLs, materials used in this study were not the same as the cigarette packaging currently in use. Also, because text warnings that matched the graphics were not available when the study was performed, the text warning area was empty. Instead, it was treated as a yellow background to indicate the text warning area. Therefore, the fixation duration on the health warning could be overestimated or underestimated. Fifth, only fixation duration was measured. In general, the major measurements used in eye-tracking research are not only fixation but also saccades. However, we focused on fixation instead of saccade, because visual fixation duration can be interpreted in the context of information processing and attention^32^. Additionally, although the use of a lower-frequency eye tracker with a sampling rate of 30 Hz could reduce the accuracy of the results^48^, we believe that it is acceptable for fixation-dependent tracking for our purpose.

## CONCLUSIONS

The GHWL attracted longer fixation durations when placed at the top or middle than at the bottom. Longer fixation durations allow images to convey information more effectively about the health risks caused by smoking, which could result in smoking cessation among smokers and induce curiosity about tobacco products and smoking in non-smokers, and hence are more effective in preventing the onset of smoking. Therefore, in countries where the GHWL is positioned at the bottom of packaging, it is recommended to move it to the top or middle. In countries where the GHWL will be introduced in the future, it should cover the top to the center of the package.
